# Health Technology Assessment Fireside: Antibiotic Prophylaxis and Dental Treatment in Canada

**DOI:** 10.1155/2013/365635

**Published:** 2012-09-02

**Authors:** Mario A. Brondani

**Affiliations:** Department of Oral Health Sciences, Faculty of Dentistry, University of British Columbia, 122/2199 Wesbrook Mall, Vancouver, BC, Canada V6T 1Z3

## Abstract

*Objectives*. This paper discusses the controversies surrounding the antibiotic prophylaxis preceding dental interventions within the following research question: how effective is dental antibiotic prophylaxis in preventing comorbidity and complications in those at risk? *Methods*. A synthesis of the available literature regarding antibiotic prophylaxis in dentistry was conducted under the lenses of Kazanjian's framework for health technology assessment with a focus on economic concerns, population impact, social context, population at risk, and the effectiveness of the evidence to support its use. *Results*. The papers reviewed show that we have been using antibiotic prophylaxis without a clear and full understanding of its benefits. Although the first guideline for antibiotic prophylaxis was introduced in 1990, it has been revised on several occasions, from 1991 to 2011. Evidence-based clinical guidelines are yet to be seen. *Conclusions*. Any perceived potential benefit from administering antibiotic prophylaxis before dental procedures must be weighed against the known risks of lethal toxicity, allergy, and development, selection, and transmission of microbial resistance. The implications of guideline changes and lack of evidence for the full use of antibiotic prophylaxis for the teaching of dentistry have to be further discussed.

## 1. Introduction 

Antibiotic prophylaxis is understood as a preventive health measure to minimize harmful interaction between the oral bacteria in the bloodstream with matrix molecules and platelets at body sites (e.g., organs, tissues), which could lead to generalized septicemia [1]. “*Because it is not possible to predict when a susceptible patient will develop an infection, prophylactic antibiotics are recommended when these patients undergo procedures that might produce bacteremia*” [2]. A prophylactic antibiotic is then given prior to the dental procedure on the basis of its activity against oral bacteria, its toxicity, and its cost. This review paper discusses the controversies surrounding this prophylaxis as a health technology within the following research question: how effective is dental antibiotic prophylaxis in preventing comorbidity and complications in those at risk? In order to address this research question, a synthesis of the available literature on the issue under the lenses of Kazanjian's framework for health technology assessment (HTA) [3, 4] is performed.

## 2. The Framework for Health Technology Assessment 

In 2004, Kazanjian presented a framework for health technological decisions with the five overlapping components as follows [3].

- *The population at risk*: Those who would benefit from the intervention, for example, those who may be at risk of suffering health problems caused by bacteremia following a dental intervention and in need of antibiotic prophylaxis.
- *The population impact*: The effects (e.g., the balance between harm and benefit) of not having the prophylaxis, which result in problems impacting quality of life: the burden of illness (e.g., functional ability and psychosocial status). Conversely, the prophylaxis itself could also cause disability in some individuals.
- *The economic concerns*: The cost-benefit/effect and utility of the prophylaxis: do the potential side effects from the medication justify the (perceived) benefits from it?
- *The broader social context*: The implications of the prophylaxis for patients (consumers), health professionals, tax-payers, and so on. This includes the ethical, legal and political implications of the antibiotic prophylaxis within the discussion of antibiotic resistance.
- *The technology assessment*: The level of evidence that the prophylaxis alleviates and/or prevents mortality/morbidity following dental treatment for those at risk.

## 3. Background: Dental Care

Dental caries (e.g., tooth decay) is still a major oral health problem in most industrialized countries, affecting up to 90% of schoolchildren and the vast majority of adults. It causes oral problems such as abscesses, toothache and is the leading cause of tooth loss [5]. Although the rates of dental care utilization vary greatly, Canadians living in urban settings tend to see dentists more regularly than they see primary health care providers. In fact, approximately 70% of Canadians older than 12 years old visited a dental office one time during a year, and from these, 50% visited a dental office twice [6]. In 2009, Canadians spent almost $13 billion on professional dental care, ranked second only to cardiovascular disorders in total direct costs. Most of the payments for dental care come from private sources, either as an out-of-pocket expense or through employer-sponsored, private insurance [7]. The direct costs (as per the money spent on dental treatment) and indirect costs (as per the number of lost days of productivity at work and school) of oral diseases and their treatment remain relevant. According to the Canadian Health Measures Survey [8], 40.36 million hours/year of school and work are lost due to dental-related issues in Canada, and 2.26 million hours/year in BC alone. The direct and indirect costs of oral disease treatment place a high burden on society, whereas the impact on quality of life resulting from these diseases can be measured by an array of self-reported questionnaires [9], which are beyond the scope of this report. Given the high prevalence of dental problems and the seeking care behavior, the risks of bacteremia can be considered in those at risk.

## 4. Population at Risk

**Figure 1:**
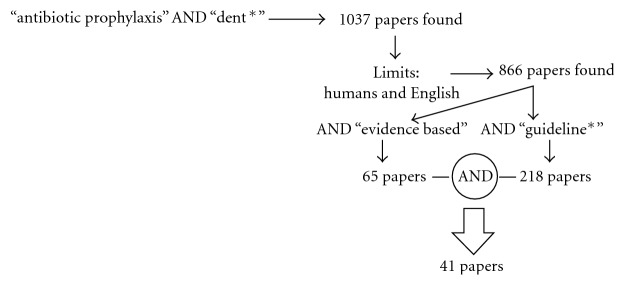
Searching strategy using PubMed and inclusion and exclusion criteria for papers.

Bacteria from the mouth can readily enter the bloodstream during daily oral hygiene activities like brushing and flossing (Table 1). The potential for bacteremia from dental procedures is more severe, however, especially when these procedures produce significant oral bleeding and/or exposure to potentially contaminated tissue such as during dental extractions, oral surgery, subgingival scaling and the subgingival placement of dental dam clamps, restorations or orthodontic bands. The bacteremia from daily oral hygiene and dental procedures does not cause harm to the majority of healthy individuals. However, those at risk of infection might develop health complications and thus might benefit from antibiotic prophylaxis. As per the population at risk from Kazanjian's framework, the author conducted a brief systematic review to discuss the guidelines used to inform dental practitioners on this health technology (HT) via PubMed using the key words “antibiotic prophylaxis” AND “dent∗” in titles, abstracts and text. 1037 results (paper titles) published since 1961 were found. A quick scan on the first 50 titles revealed that some were related to *in vitro* studies while others were in languages other than English. These 1037 titles were limited to “humans” and published in English, which lead to 886 titles: *(((antibiotic prophylaxis) AND dent*∗*) AND human) AND English [Language]*, see Figure 1.

When 886 titles were limited to “evidence based,” 65 papers were found. When the same 886 titles were limited to “guidelines,” 218 papers were found. After combining these two sets, 41 publications were identified as potential evidence-based guidelines. Six were excluded because there was no full text available, and four because they were not related to dentistry. From the 31 publications left, two were erratum from Wilson's et al. work [10, 11], and four were the same paper published four times entitled “Prevention of bacterial endocarditis: recommendations by the American Health Association.” This paper was originally published by Dejani and colleagues at the Journal of the American Medical Association in June 1997 and again at Circulation in July 1997, American Dental Association in August 1997, and Clinical Infections Disease in December 1997. Within the 28 papers left, six referred to guidelines developed in Australia [12], Sweden [13], or the UK [14]. The 20 papers left are presented throughout this report while others have been added to support and refute arguments presented.

## 5. Understanding the Existing Guidelines 

The development of guidelines for antibiotic prophylaxis has been driven by the perceived needs of those considered to be “at risk.” Until recently, those considered “at risk” would include individuals having one or more of the following: cardiac deformities, total joint replacement, or a weakened immune system [15]. Not long ago, this list also included individuals with diabetes type I, malnourishment, and hemophilia.

It seems that the first recommendation for giving antibiotic prophylaxis to patients undergoing dental procedures was put forward by the American Heart Association in 1955. It was only in 1990 that such recommendations became an actual guideline adopted by the American Council on Clinical Affairs [16]. Since then, the guidelines have been revised on several occasions: in 1991, 1997, 1999, 2002, 2005, 2007, 2008, and lately in 2011 [2] in accordance with, among other things, the idea that the harms of antibiotic-associated adverse events may exceed the benefit, if any, and also because of the increasing development of resistant strains of bacteria [10, 11]. 

In this regard, the American Council on Clinical Affairs (ACCA) made changes to the guidelines [2] based on dental and medical literature pertaining to postprocedural bacteremia-induced infections. This included a systematic literature search of PubMed with the following key words: infective endocarditis (IE), bacteremia, antibiotic prophylaxis, and dental infection. The ACCA gathered publications within the last 15 years pertaining to humans and involving clinical trials. One hundred and thirteen articles matched these criteria and were amalgamated by recommendations from experts and/or consensus opinion of experienced researchers and clinicians (which is considered a low level of evidence) [14]. The ACCA also revised the “Prevention of Infective Endocarditis: Guidelines from the American Heart Association” (AHA). 

The Council recommended the conservative use of antibiotics to minimize the risk of developing resistance to current antibiotic regimens. They called for judicious use of antibiotics for the prevention of IE while keeping in mind that the awareness of the potential relationship between IE and dental treatment dates back to 1909, [17] a time when infection control and sterilization were not well developed and understood. 

The Council on Clinical Affairs review is very similar to the work undertaken by Wilson and his 22 team members [10, 11] and endorsed by the Council on Scientific Affairs of the American Dental Association, the American Academy of Pediatrics, the Infectious Diseases Society of America, the International Society of Chemotherapy for Infection and Cancer, and the Pediatric Infectious Diseases Society. Wilson's group recommendations were based on the analyses of relevant literature regarding: procedure-related bacteremia and infective endocarditis, *in vitro* susceptibility of infective endocarditis causing microorganisms, results of prophylactic studies in animal models, and retrospective and prospective studies of the prevention of infective endocarditis. They used MEDLINE database searches from 1950 to 2006 for English-language papers with the following key-words: endocarditis, infective endocarditis, prophylaxis, prevention, antibiotic, antimicrobial, pathogens, organisms, dental, gastrointestinal, genitourinary, streptococcus, enterococcus, staphylococcus, respiratory, dental surgery, pathogenesis, vaccine, immunization, and bacteremia. They also searched the reference lists of the identified papers. Although they based their arguments on most of the papers that would be included within the Clinical Affairs review three years later, they concluded that only an extremely small number of cases of infective endocarditis would be prevented by antibiotic prophylaxis for dental procedures even if such prophylactic therapy was 100% effective. Such conclusions were not explicitly stated at the Council's review. 

As seen in the above discussion, the evidence for using antibiotic prophylaxis to prevent IE alone seems weak to say the least. Although the many revisions to the existing guidelines attest to the weakness of this association, the existence of so many different and yet overlapping guidelines is frustrating at best and confusing at worse. 

A similar situation happened in Canada. In February of 2005, the Canadian Dental Association (CDA) issued a position statement emphasising that patients at risk would include those under the following descriptions [18].

1. Patients with *cardiac deformities* and/or *artificial devices in the circulatory system* should receive antibiotic prophylaxis according to the current guidelines of the AHA. Consultation with the patient's physician may be required.
2. Patients with a variety of *immunocompromising conditions, such as those with HIV/AIDS, those who have had organ transplants, and those undergoing cancer treatment*, should receive antibiotic prophylaxis using the current protocols of the AHA. Such patients would include those with a suppressed leukocyte count where the white blood cell count (WBC) is less than 3500 cells/mm^3^ (3.5 K/mm^3^) or the absolute neutrophil count (ANC) is less than 500 cells/mm^3^ (0.5 K/mm^3^).
3. Patients with *total joint replacement* (not those with only pins, screws, and/or plates) should receive prophylactic antibiotics as well as those with recent (within two years) joint replacement or previous prosthetic joint infection. Consultation with the patient's orthopedic surgeon may be required.

Of interest from the three above points, the CDA only recommended consultation with the patients' health care provider in two, not for cases involving immunecompromising conditions. On July 3, 2007, the CDA [15] updated its position statement by issuing an electronic communication to the dental profession saying that *“in light of these new guidelines, CDA has now rescinded its 2005 position on Antibiotic Prophylaxis for Dental Patients at Risk. The withdrawn statement addressed the needs of patients at risk of infective endocarditis and those with total joint replacement. To ensure that Canadian dentists are not left without guidance on those topics while new material on antibiotic prophylaxis is being produced by its Committee on Clinical and Scientific Affairs, CDA has endorsed the new AHA guidelines and maintains its endorsement of the 2003 statement of the American Dental Association and the American Academy of Orthopaedic Surgeons concerning antibiotic prophylaxis for dental patients with total joint replacements.”* The 2003 guideline was not found. The communication also had links to the AHA (New guidelines regarding antibiotics to prevent infective endocarditis) and to the American Dental Association—ADA and the American Academy of Orthopaedic Surgeons—AAOS (Antibiotic prophylaxis for dental patients with total joint replacements). Unfortunately, the links were not functioning (*“page not found”*).

In November of 2007, the CDA issued another letter to again maintain its support of the 2003 statement of the ADA, but to say also that routine antibiotic prophylaxis *“was no longer”* indicated for most dental patients with total joint replacements, nor for patients with orthopedic pins, plates, and screws [15].The statement rationale was that patients should be in optimal oral health prior to having total joint replacement and should maintain good oral hygiene and oral health following surgery. However, the statement made it clear that when orofacial infections in patients with total joint prostheses are happening, they should be treated rigorously to prevent its spread. It also reemphasised that prophylactic antibiotics would still be considered for a small number of patients who

- are within the first two years following joint replacement,
- are immunocompromised/immunosuppressed, and
- have had a history of prosthetic joint infections.

In December 5, 2008, the ADA updated its guidelines to say that patients who have taken prophylactic antibiotics in the past with the following conditions no longer need them: mitral valve prolapsed, rheumatic heart disease, bicuspid valve disease, calcified aortic stenosis, congenital heart conditions such as ventricular septal defect, atrial septal defect, and hypertrophic cardiomyopathy [19]. The ADA stated that these revisions were based on scientific evidence attesting that the harms of taking preventive antibiotics outweigh the benefits for most patients. Interestingly, the November 2007 position paper from the CDA states that “*these recommendations are based upon a variety of in vitro studies, clinical experience, animal model data and an assessment of the common oral flora most likely to cause potential bacteremias. Definitive patient risk/benefit ratios for these prophylactic procedures have not been determined nor have they been medically or scientifically proven to be effective by well-designed controlled human trials (with or without randomization).*” Both 2008 ADA and 2007 CDA statements questioned the use of antibiotic prophylaxis. However, the CDA stated that such a conclusion has not been scientifically proven while the ADA mentioned that it was scientifically based. A closer look at both statements reveals that they have very few references while offering email and a phone number in case the reader has any questions about these recommendations. The CDA statement even cautions that *“this information was created by the Canadian Dental Association for use by CDA member dentists. It should not be used as a replacement for professional dental or medical advice*.*”*

On an ADA webpage entitled “Oral Health Topics” [19], two links are offered, one for dentists, the other for patients. The dentists' link says that the available information is mixed as to whether or not prophylactic antibiotics taken prior to a dental procedure actually prevent IE. The recommendation brings back the notion that people who are at risk for IE are regularly exposed to oral flora during basic daily activities such as brushing or flossing, suggesting that IE is more likely to occur as a result of these everyday activities than from a dental procedure that may happen only once. It goes on to say that “*the ADA and the AAOS are currently in the process of developing evidence-based clinical guidelines on the topic of antibiotic prophylaxis for patients with orthopedic implants undergoing dental procedures.”* Although this evidence-based clinical guideline is yet to be seen, the patients' link uses lay language to discuss the risks of this HT in regard to IE and the actual need for it:

- the risks of adverse reactions to antibiotics (form mild rashes to severe breathing problems that could result in death) outweigh the benefits of prophylaxis for most patients;
- when all the study results are looked at together, it is not clear that premedication prevents IE;
- bacteria from the mouth can enter the bloodstream during daily activities like brushing or cleaning between the teeth. People at risk of infection might be more likely to develop IE from these activities than from a dental treatment.

In all guidelines and papers on this HT, the one message that comes across is that the dental professionals must consider the potential benefit of antibiotic prophylaxis versus the risks of adverse reactions for each patient [2, 15, 20]. In fact, Bach (2010) highlighted that these guideline modifications reflect a change in recommendations prompted by a change in philosophy despite the lack of new data. The author goes on to say that, to some degree, the arguments for and against antibiotic prophylaxis become those of philosophy, ethics, and the role of evidence-based medicine [21]. 

With no surprise, the Cochrane Collaboration on antibiotics for the prophylaxis of bacterial endocarditis in dentistry (as revised in 2008) concluded that the implication for practice is that such HT is of unknown effectiveness. They based their argument by saying that *“no reliable evidence was found to determine whether antibiotic prophylaxis is effective or ineffective against bacterial endocarditis in people at risk, who are about to undergo an invasive dental procedure”* [22]. However, this finding is not new. Clements and Ransohoff [23] almost 20 years ago had already concluded there was a very small risk of IE (4.1 cases per 1 million dental procedures), which was outweighed by a greater risk of fatal reactions to antibiotics such as penicillin as still one of the most recommended class of antibiotics (15 deaths per 1 million procedures). Similarly, Uçkay and colleagues [24] conducted a PubMed review of 144 papers on the use of dental antibiotic prophylaxis in patients with total joint replacement and concluded that this HT was not recommended given the lack of evidence-based information. Although they did suggest the prophylaxis for those patients within the first year of the total joint replacement, they did not support the 2-year period as suggested by the CDA 2007 guideline. And the guidelines are not the same throughout the world [12, 13, 25], which might imply a different philosophy of practice and a different understanding of risks since the dental procedures causing bacteremia are the same. It might also demonstrate that the guidelines have not always been reviewed at the same pace worldwide [26]. 

## 6. Population Impact

It appears that we have been using this HT without a clear understanding of its benefits, and this has been driven mostly by the possibility of infective endocarditis and infection of artificial joints, which have been now removed from being fully “at risk” conditions. When bacteremia happens, however, the treatment of the resulting complication can be not only costly, but have a detrimental impact on quality of life. For example, though uncommon, IE infection has an incidence rate ranging from 5.0 to 7.9 per 100.00 person-years with a significant increase among women in the USA [27]. However, the prevalence of this disease has remained approximately the same for the past 40 years with a mortality rate between 15% and 30% despite advances in antimicrobial therapy and cardiovascular surgery. As per the total joint placement, between 450,000 to 1 million procedures are performed annually in the USA [27], and about 50.000 in Canada [28]. In terms of cost, hip replacement alone adds up to 15 billion/year in the USA [29]. If these joints get infected due to dental work or any other invasive medical intervention or surgery, the procedure is considered a failure and the need for extensive and costly additional treatment follows [20]. In terms of prevalence of immunecompromised conditions, Kahn (2008) estimated that more than 10 million individuals in the USA would be considered as having their immune system compromised mostly due to having HIV/AIDS, being organ transplant recipients, or being in the midst of cancer treatment [30]. There was no paper showing evidence on the financial consequences to the health care system of a patient not having antibiotic prophylaxis and so developing IE or total joint replacement infection after a dental intervention per se. 

## 7. What Is the Incidence of Bacteremia-Related Events Following Dental Procedures?

There are only few studies showing the incidence of bacteremia after different dental procedures. Hall and colleagues (1999) [31], for example, presented a summary from various studies to show that the incidence can vary between 5% and 97%, depending on the procedure, including regular brushing which has a rate of 25% of the time (Table 1). But how much of these are actually worrisome to patients at risk is yet to the found. One factor to be considered is the duration of the dental intervention since positive blood cultures can be identified as earlier as in 30 seconds after the procedure started [32, 33]. In this case, however, it may be difficult to estimate the exact time-frame as some studies have found that the shorter the appointment, the higher the incidence of positive blood cultures,[34] whereas others were inconclusive [35]. 

As the Clinical Affairs Committee stated, the impossibility of predicting when a susceptible patient will develop an infection from these potential sources of bacteremia warrants prophylactic antibiotics when these patients undergo dental procedures [2]. However, the latest available recommendations do not support this view. Considering that bacteremia can occur from simply brushing, and that people brush their teeth at some point, the actual risk for bacteremia specifically after some dental procedures remains unknown. Wilson and colleagues stressed the fact that the vast majority of cases of infectious endocarditis caused by oral bacteria result from bacteremia associated with routine daily activities such as tooth brushing, flossing, and chewing. Given the frequency in which these events happen daily, what are the economic concerns to support antibiotic prophylaxis? 

## 8. Economic Concerns

One of the major drivers of antibiotic prophylaxis has been to avoid burdening the health care system with treatment costs for conditions stemming from the presence of oral bacteremia. On that point, the American Academy of Orthopaedic Surgeons stated that *“given the potential adverse outcomes and cost of treating an infected joint replacement, [we] recommend that clinicians consider antibiotic prophylaxis for joint replacement patients prior to any invasive procedure that may cause bacteremia* [27].*”* The same could be easily said about those at risk for any medical complication arising from bacteremia, although the CDA does not fully support such a recommendation. In one paper discussing the cost-effectiveness of IE associated with dental procedures, the authors evaluated the number of IE cases prevented and years of life saved [36]. They concluded that, optimistically, oral amoxicillin prophylaxis would prevent 32 cases of IE per million dental procedures at the approximate cost of $119,000 per prevented case and $21,000 per year of life saved. Erythromycin prophylaxis was slightly less expensive per benefit than amoxicillin because of lower cost and lack of drug anaphylaxis. Sensitivity analyses suggested that erythromycin prophylaxis might be cost-saving. Thus, the paper concludes that using oral antibiotics to prevent IE is reasonably cost-effective when looking at cumulative morbidity and incremental health care costs. However, using another population age group, Caviness and colleagues concluded the opposite [37]. When performing an analysis of the cost-effectiveness of antibiotic prophylaxis for bacterial endocarditis in children aged 0–24 months, the authors found that prophylaxis would prevent 7 bacterial endocarditis cases per 1 million children treated. But when antibiotic-associated deaths were included, the no-prophylaxis strategy was more effective and less costly than the prophylaxis strategy. When antibiotic-associated deaths were excluded, amoxicillin cost $10 million per Quality-Adjusted Life Years gained and $70 million per case prevented. So, prophylaxis was not a cost-effective use of health care resources for children [37]. 

Although there was no similar type of cost-effective analysis for immunocompromised individuals, Jacobson et al. (1991) [38] performed a decision-utility modeling analysis on the associated costs of antibiotic prophylaxis for 1 million patients with prosthetic joint replacements who were hypothesised as having invasive dental procedures. In the model in which no patients were given antibiotics, the number of joints with bacteremia would be 30 per million with almost 2 deaths and 3 amputations at a total cost of $2.29 million dollars. In the model where all patients were given penicillin/amoxicillin prophylaxis, the number of deaths would increase to 2.3 with the possibility of 400 cases of anaphylaxis at a total cost of $6.4 million dollars. Like those patients with IE, patients with joint replacements seem not to benefit from this HT. 

## 9. Misuse of Antibiotics and Side Effects

Antibiotic prophylaxis usually involves a single dose of antibiotic often given to the patient close to the time of the appointment and differs from treatment that entails a course of antibiotics over a period of time. The oral, intramuscular, or intravenous recommended doses of antibiotics to be taken by those at risk before dental appointments are well known and do not seem to have changed [2]. Dentists can make use of three broader families of antibiotics: *Beta-Lactams* that are narrow spectrum bactericidals, which inhibit the building of the bacterial cell wall by interference with the synthesis of peptidoglycan (Amoxicillin as an Aminopenicillin, and Cephalexin/Cephazolin as a Cephalosporin) and *Lincosamides* (Clindamycin) and *Macrolides* (Claritromycin) that are narrow spectrum bacteriostatics, which inhibit protein synthesis at the ribosomal level. According to some studies, there has been an inadvertent use of antibiotics. Babbour and coworkers revised papers on antibiotic resistance [39] specifically with regards to the treatment of IE and highlighted cases of multidrug resistance among viridans group streptococci, Vancomycin- and Oxacillin-resistant Staphylococcus aureus, Vancomycin- and Aminoglycoside-resistant enterococci. Aside from antibiotic resistance, side effects of antibiotics are many [2]. Amoxicillin is well known for its potential allergic reactions (rash, fever, eosinophilia) and anaphylactic shock to those allergic. Patients with mononucleosis might develop maculopapular exanthema while on Amoxicillin. Cephalexin and Cephazolin might have these side effects plus thrombocytopenia. Clindamycin can be associated with the development of pseudomembranous colitis by *Clostridium difficile*, and gastrointestinal disorders. Claritromycin might also cause gastrointestinal disorders, allergic reactions, and liver damage and have to be used with caution during pregnancy [40]. Even more worrisome is the association between antibiotic consumption and an increased risk of cancer, [41] including breast cancer (women taking more than 25 antibiotic prescriptions or more than 500 days over 17 years have double the incidence of this malignancy) [42]. These findings set the stage to discuss the broader social context of antibiotic prophylaxis. 

## 10. The Broader Social Context: The Case of Canada

Although any estimate of antibiotic consumption in Canada is difficult to make, Hutchinson and colleagues (2004) [43] believe that the Defined Daily Dose per 1000 inhabitant-days is about 18 for British Columbians, 50% more than the Danish and 80% more than the Finnish [41]. Antibiotic consumption per capita seems to be 20% higher elsewhere in Canada compared to Quebec [44]. Not surprisingly, news media reports have emphasised that Canadians (and most of North Americans) are becoming increasingly resistant to antibiotics, getting sicker more frequently, and taking longer to recover. Information on this topic can be gathered from websites, media campaigns and books [45]. Thus the lay public is engaging in discussion of, or at least thinking about, the indiscriminate use of antibiotics. Recently, Patrick and Hutchinson (2009) [46] suggested “the medical profession to engage governments to assist in striking the best balance between controlling antibiotic use through formulary restrictions and making antibiotics available to those who can truly benefit.” However, entrenched prescriptions habits and patient expectations are hard to change. In Australia, as many as 50% of antibiotic regimens prescribed are believed to be inappropriate [12]. Within the public arena, searching sites such as Google, which may get 2.5 billion searches everyday, displays an array of media information that is taken at face value by the public regardless of its scientific evidence. For example, a google search using the words “antibiotic, dentist, prescribe” brings more than 5 million hints (as per March 29th, 2012, from the authors' personal computer at the UBC Faculty of Dentistry), and the second listed hint links to a blog from a medical doctor dissing the dental colleagues (http://mdwhistleblower.blogspot.ca/2010/05/why-do-dentists-prescribe-prophylactic.html). Although the blog does discuss the role of the physicians on this issue, the emphasis is that “dentists irrationally prescribe antibiotics.” And this is what the lay public reads.

## 11. Effectiveness of Evidence—Technology Assessment

In order to prevent bacteremia, an appropriate dose of a prophylactic antibiotic should be given prior to the procedure so that an effective tissue concentration of the drug is present to protect the patient from a bacteremia-induced periprosthetic sepsis [27]. However, as discussed above, prophylaxis may prevent an exceedingly small number of cases of IE and bacteremia in general, if any, in individuals who undergo a dental, GI tract, or GU tract procedures. As in any therapeutic use, the use of antibiotics for prophylaxis carries a risk of adverse drug reactions as discussed above [20]. In its latest 2012 issue [47], the Journal of the CDA restated its November 2007 position on not recommending antibiotic prophylaxis for patients who have had total joint replacement. It also refers to the work from Thornhill and colleagues from the U.K. National Institute for Health and Clinical Excellence (NICE), [25] which no longer recommends this HT for patients at risk for IE, which has been previously stated by the Cochrane Review and others. In fact, after this recommendation took place in the U.K., there was no significant increase in the number of IE cases. Interesting to note is that before this recommendation, more than 90% of all antibiotic prescriptions were given by dentists, and this dropped from 10,727 prescriptions/month to 2,292 prescriptions/month after the NICE report in the UK. 

## 12. Conclusions—How Effective Is Dental Antibiotic Prophylaxis in Preventing Comorbidity and Complications in Those at Risk?

Kazanjian's framework offered an alternative view on the issue of antibiotic prophylaxis as a health technology in the light of its Economic Concerns, Population Impact, Social Context, Population at Risk, and the Effectiveness of the evidence to support its use. With focus on the latter, the guidelines and papers discussing this HT have emphasised that any perceived potential benefit from administering antibiotic prophylaxis before dental procedures must be weighed against the known risks of lethal toxicity, allergy, and development, selection, and transmission of microbial resistance. Although evidence-based clinical guideline is yet to be seen, one thing to keep in mind is that they are guidelines, and variation might occur as dentists must exercise their clinical judgment, communicate effectively with patients and other health care providers, and respect patients' autonomy in determining whether or not antibiotic prophylaxis is appropriate [48, 49]. In fact, the NICE guidelines [25] state clearly that *“treatment and care should take into account patients' needs and preferences. Patients should have the opportunity to make informed decisions about their care and treatment.”* It is worth mentioning that population at risk, in epidemiological terms, relates to those who would benefit the most from the intervention (e.g., HT). This population, as discussed above, has *changed* throughout the years, from those with cardiac deformities, total joint replacement, weakened immune systems, diabetes type I, malnourishment and hemophilia to very specific cases in which professional judgment is deemed important. More often than not, however, patients prefer to take the prophylaxis as they perceive it as “safer” if they think complications from dental treatments are life-threatening (unlikely), without (or not wanting to have) full understanding of the risks of antibiotic use. The implications of such guideline changes and lack of evidence for the full use of antibiotic prophylaxis for the teaching of dentistry have to be further discussed. 

## Figures and Tables

**Table 1:** Incidence of bacteremia in percentage following oral and dental procedures.

Procedure	Incidence (%)
Suture removal	5
Brushing teeth	25
Rubberdam placement	29
Matrix band placement	32
Dental scaling	35
Mastication	38
Oral extraction	40
Endodontic treatment	42
Periodontal surgery	58
Intraligamental injection	97

^*^The incidence shown refers to multiple studies and varies widely. Adapted from Hall et. al. (1999) [31].
